# Mapping the relationship between obesity and endometrial cancer: current research hotspots and future trends (2003-2024)

**DOI:** 10.3389/fonc.2025.1567022

**Published:** 2025-10-02

**Authors:** Lei Liang, Chuangxiu Song, Bo Yang, Chun Chang, Shichao Chen, Li Sun

**Affiliations:** ^1^ The 980 Hospital of the Joint Logistic Support Force of the Chinese People’s Liberation Army, Shijiazhuang, Hebei, China; ^2^ Neck-Shoulder and Lumbocrural Pain Hospital of Shandong First Medical University, Jinan Shandong, China

**Keywords:** obesity, endometrial cancer, research hotspots, research trends, bibliometric analysis

## Abstract

**Background:**

Obesity is an independent risk factor for endometrial cancer (EC). Bibliometrics allows for the analysis of multiple data from published publications to identify the current state of research and future trends and to construct a knowledge framework. There is a lack of high-quality bibliometric analyses of obesity and EC.

**Methods:**

This study retrieved publications related to obesity and EC from the Web of Science Core Collection (WOSCC) from 2003 to 2024. Publication trends were analyzed using Microsoft Excel 2019, while CiteSpace (v.6.4.R1 Advanced) was employed to analyze institutional co-occurrence, cited journals, journal co-citation mapping, co-cited references, and keywords. VOSviewer (v.1.6.20) was used to analyze the journals in which the publications appeared. SCImago Graphica (v.1.0.39) was utilized to investigate the distribution and collaboration of countries/regions, institutional collaborations, and author collaborations.

**Results:**

681 publications from 2003 to 2024 were included in the final analysis. The volume of publications showed an upward trend, peaking in 2021. The United States was the country with the highest number of publications, with the National Cancer Institute (NCI) being the leading institution. Scholars Emma J. Crosbie and Faina Linkov had the highest publication counts, while CALLE EE was the most cited scholar. The journal Gynecologic Oncology (Q1/4.5) published the most relevant articles and was also the most frequently cited journal. The most common keywords were “endometrial cancer,” “body mass index,” and “risk.” Current research focuses on exploring the mechanisms linking obesity and EC and analyzing the impact of obesity on clinical treatment strategies for EC. Future research directions include: (1) expanding the scope to related diseases of EC; (2) emphasizing typical indicators and diagnostic techniques for EC; (3) developing new treatment methods and technologies to enhance clinical efficacy; and (4) further strengthening the exploration of the pathological mechanisms related to obesity and EC.

**Conclusion:**

This study comprehensively summarizes the knowledge structure of obesity and EC and identifies key research hotspots and trends. Based on our findings, the formation of a multidisciplinary team, the rational application of diagnostic and therapeutic techniques, the further enhancement of the exploration of the pathological mechanisms associated with obesity and EC as well as the improvement of clinical diagnostic and therapeutic strategies are powerful measures to promote the development of this field.

## 1 Introduction

Endometrial cancer (EC) is a common gynecological tumor, primarily affecting women in the menopausal and perimenopausal stages (1, 2). Data indicates that EC accounts for 1% of global mortality, with a lower mortality rate compared to other cancers (3). Since 2005, the incidence and mortality rates of EC have gradually increased, with an annual change in incidence of 5% (4, 5). It is projected that by 2030, EC will surpass colorectal cancer, becoming the fourth leading cause of cancer-related deaths among women (3, 6).

Currently, over half of EC cases can be attributed to obesity (7), which is not only an independent risk factor for EC but also a determinant of its progression and treatment outcomes. The relationship between obesity and EC has been established, following a dose-response pattern where the incidence of EC increases with rising body mass index (BMI) (8, 9). Research shows that among the 20 most common cancers, EC has the strongest association with obesity; for every 5 kg/m² increase in a patient’s BMI, the risk of developing cancer increases by 5.4% (10), with women having a BMI greater than 40 kg/m² facing a 10-15% risk of developing EC (11). Obesity also negatively impacts the mortality rate of EC; a prospective study revealed that obese patients with early-stage EC had higher mortality rates compared to women with normal BMI, with 67% of these deaths attributed to obesity-related causes (12).

Currently, obesity is becoming increasingly prevalent worldwide, with a more pronounced rise in obesity rates among women (13), which has become a driving factor for the increasing incidence of EC. Studies have found that obesity creates a pro-inflammatory environment characterized by elevated circulating levels of C-reactive protein, interleukin-6, and tumor necrosis factor, which may be the mechanism by which obesity increases the risk of EC (14). Inflammatory cells induce rapid cell division and produce increased concentrations of free radicals and may cause damage to DNA, increasing the likelihood of converting DNA damage into mutations (15, 16).It has been demonstrated that the molecular pathways associated with cancer inflammation involve the nuclear factor-κB transcription factor and its inhibitor, the κB kinase complex (17).

Despite current research confirming the close link between obesity and EC, and the consideration of obesity’s impact on screening, diagnosis, and treatment protocols for EC, there has yet to be a systematic review of the relationship between obesity and EC. Bibliometric analysis, which examines published academic literature to describe publication trends and relationships among these works, can assess the impact of published works on future research in the health field through the analysis of various data (18, 19). To the best of our knowledge, this is the first application of bibliometric analysis of obesity and EC. The comprehensive analysis of authoritative data helps us to get informative findings. This is of positive significance for further recognizing the association between obesity and EC, exploring the pathological mechanisms between the two as well as improving diagnostic methods and treatment strategies.

## 2 Materials and methods

### 2.1 Data sources and search strategy

WOSCC database is an influential multidisciplinary academic literature abstract indexing database in the world, which includes more than 22,000 authoritative and high-impact academic journals in the world. The citation information and data analysis tools provided by WOSCC are important references for scholars to conduct academic research and academic evaluation.Therefore, we choose WOSCC as the source of literature search.We searched the WOSCC database to gather relevant publications, with the search timeframe extending from the inception of the database to January 9, 2024. We employed search terms related to obesity and endometrial cancer for thematic retrieval. The specific search strategy was: (“obesity” OR “obese*” OR “overweight”) AND (“Endometrial Neoplasm*” OR “Neoplasm, Endometrial” OR “Neoplasms, Endometrial” OR “Endometrial Carcinoma” OR “Carcinoma, Endometrial” OR “Carcinomas, Endometrial” OR “Endometrial Carcinomas” OR “Cancer of Endometrium” OR “Endometrium Cancer*” OR “Cancer, Endometrium” OR “Cancers, Endometrium” OR “Cancer of the Endometrium” OR “Endometrium Carcinoma*” OR “Endometrial Cancer” OR “Cancer, Endometrial” OR “Cancers, Endometrial” OR “Endometrial Cancers”). The retrieved publications were exported in plain text format to Endnote X9.3.3 data management software, selecting the data record format as “full record and reference citations”.

### 2.2 Data processing and analysis

**Figure 1:**
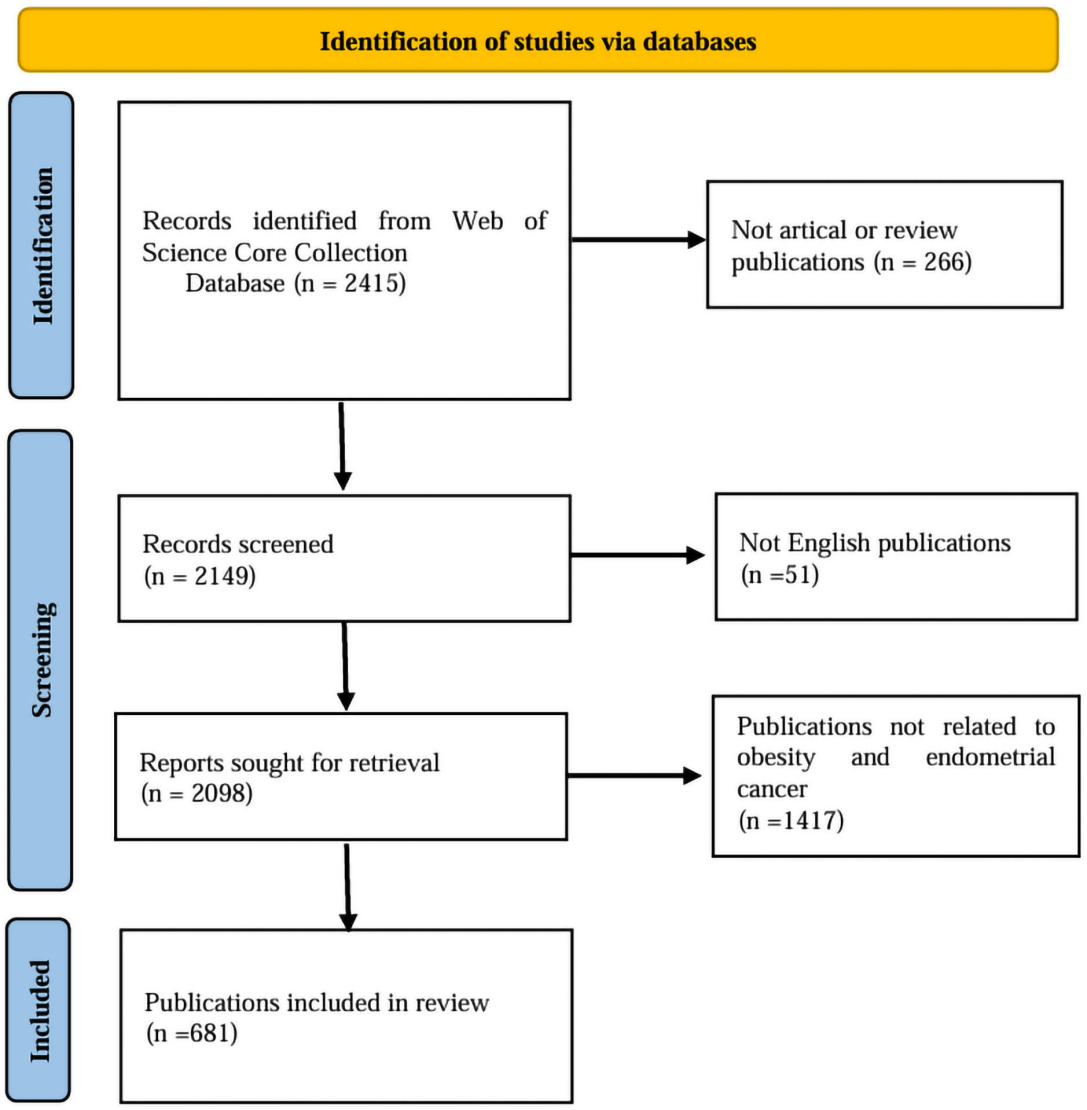
Literature screening process.

To ensure the accuracy of the data analysis results, we conducted a selection of the retrieved publications. Initially, we utilized EndNote X9.3.3 software to eliminate duplicates, followed by a manual review of titles and abstracts to filter publications that met the inclusion criteria. This process was carried out independently by two researchers (SCX and YB), with any discrepancies resolved by a third researcher (CC). The inclusion criteria for publications were: (1) the type of publication must be either an ARTICLE or a REVIEW; (2) publications must be in English; (3) there were no restrictions on the species studied, allowing for both animal models and human research. The exclusion criteria were: (1) publications unrelated to the research topic; (2) publications for which the full text was unavailable. A total of 681 documents were screened, comprising 579 articles and 102 reviews. **Figure 1** illustrates the specific screening process. The data analysis of the selected publications was conducted by a researcher (CSC) who operated the software independently.

Employ Microsoft Excel 2019 to examine publication trends, while CiteSpace (v.6.4.R1 Advanced) will be used to analyze institutional co-occurrences, cited journals, journal overlay mapping relationships, co-cited references, and keywords. VOSviewer (v.1.6.20) is designated for analyzing publishing journals. SCImago Graphica (v.1.0.39) will facilitate the analysis of national/regional distribution and collaboration relationships, institutional collaborations, and author collaborations.

We have configured the parameters by reference to the research and methodology of Professor Chen Chaomei, founder of the CiteSpace (v.6.4.R1 Advanced)(20). The specific settings are as follows: Period: 2003-2024; Slice Length: 1 year; Selection Criteria: gindex(k=25); Pruning: pathfinder, pruningsliced networks. After the setup, different analytical contents were visualized and analyzed as network nodes. To capture rapid, intricate evolutionary dynamics within the research domain and avoid potential loss of critical details that longer time slices might entail, the temporal resolution was set to one year. When k=25, the most lucid and information-rich network maps emerge, circumventing both the omission of vital nodes due to excessively low k values and the obscuring of core structures caused by overly dense networks resulting from excessively high k values. In keyword analysis, when synonyms appear, keyword merging should be performed. The specific merging method is as follows: (a) Select the primary keyword: Click the primary keyword to be retained and choose ‘add to alias list (primary)’. (b) Select secondary keywords: Click the secondary keywords to be removed and select ‘Add to alias list (secondary)’. (c) Regenerate the graph: Close the existing visualisation interface and click the ‘Go’ button again to complete the merging process.

The main operational steps for VOSviewer include: creating a map based on bibliographic data, importing source data, selecting analysis options, and setting appropriate thresholds to generate the network view. Different thresholds should be selected according to varying analytical items. However, threshold selection must adhere to the following criteria: (a) avoid an insufficient number of nodes that would result in inadequate image information and prevent network relationship analysis; (b) avoid an excessive number of nodes that would render image information overly complex and hinder the formation of thematic relationships; (c) ensure the resulting image highlights key elements and achieves a favourable visual effect.

The primary parameters for SCImago Graphica (v.1.0.39) software are set as follows: Size: frequency; Color: clusters; Label: choose according to the content of the analysis, using the same color as marks; Layout: Circular; Edges: use the same color as marks.

## 3 Results

### 3.1 Trends in the growth of publications

**Figure 2:**
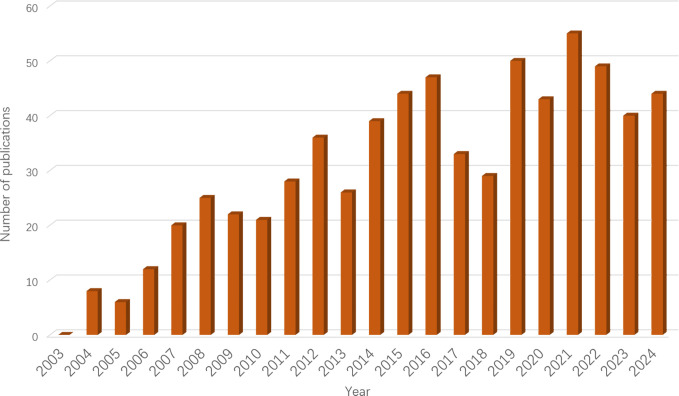
Annual trend of publication.

**Figure 2** illustrates the annual publication volume. The 681 articles included in this analysis were published between 2003 and 2024, showing an upward trend in publication numbers, peaking in 2021. The publication count experienced four significant milestones: in 2012, the number of publications first exceeded 30; in 2016, it surpassed 40; in 2019, it reached 50; and it peaked in 2021. Although there was a decline in publication volume from 2022 to 2024, the count remained no less than 30, indicating that research on the relationship between obesity and endometrial cancer continues to be a focal point of interest among scholars.

### 3.2 Distribution and collaboration by country/region

**Figure 3:**
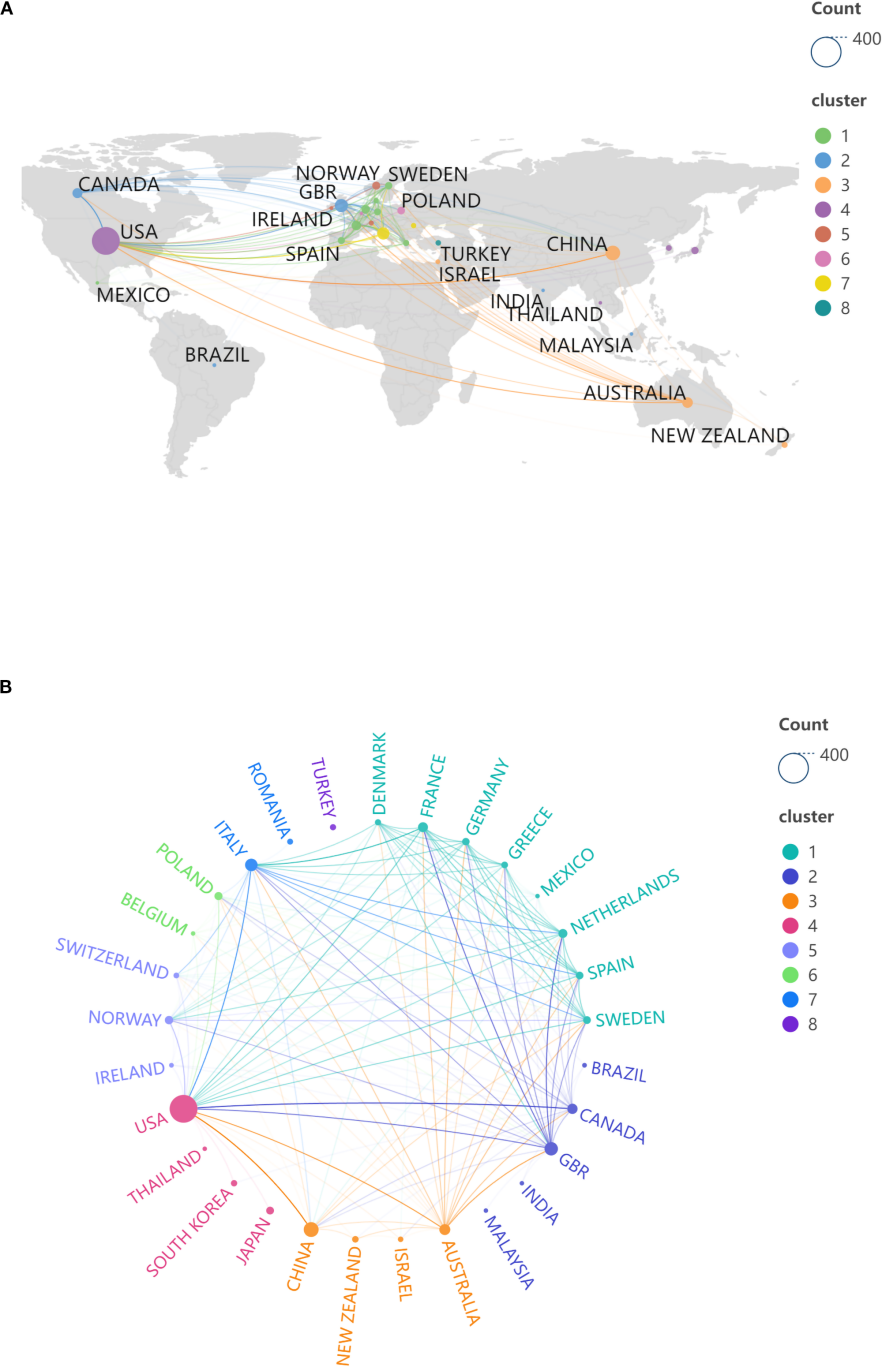
Country/region analysis view. **(A)** View of the geographical distribution of countries/regions; **(B)** Country/regional partnership view.

A total of 266 countries or regions have contributed to publications on this topic. **Table 1** presents the top 15 countries ranked by the number of publications, each having published no fewer than 15 articles. **Figure 3A** illustrates the geographical distribution of these countries. The results indicate that relevant research on this topic is widely prevalent across the globe. The United States and China are the primary contributors to this research area, with 302 and 84 publications, respectively, leading other countries or regions. This underscores the significant investment of resources and effort by the United States and China in this field of study.

**Table 1:** Top 15 countries by the number of publications.

Rank	Count	Country
1	302	USA
2	84	CHINA
3	68	GBR
4	59	ITALY
5	44	AUSTRALIA
6	39	CANADA
7	36	FRANCE
8	29	NETHERLANDS
9	25	NORWAY
10	23	POLAND
11	21	JAPAN
12	21	SWEDEN
13	20	SPAIN
14	19	GERMANY
15	15	GREECE

**Figure 3B** depicts the collaborative relationships among relevant countries or regions worldwide, which can be categorized into eight clusters, indicating extensive cooperation among these nations. Notably, the collaboration between the United States, China, and Australia, as well as between the United Kingdom, the United States, France, and the Netherlands, stands out. Additionally, Australia has established collaborative ties with several countries, including Italy and Canada. International collaboration plays a crucial role in the research on this topic.

### 3.3 Distribution and cooperation between institutions

**Figure 4:**
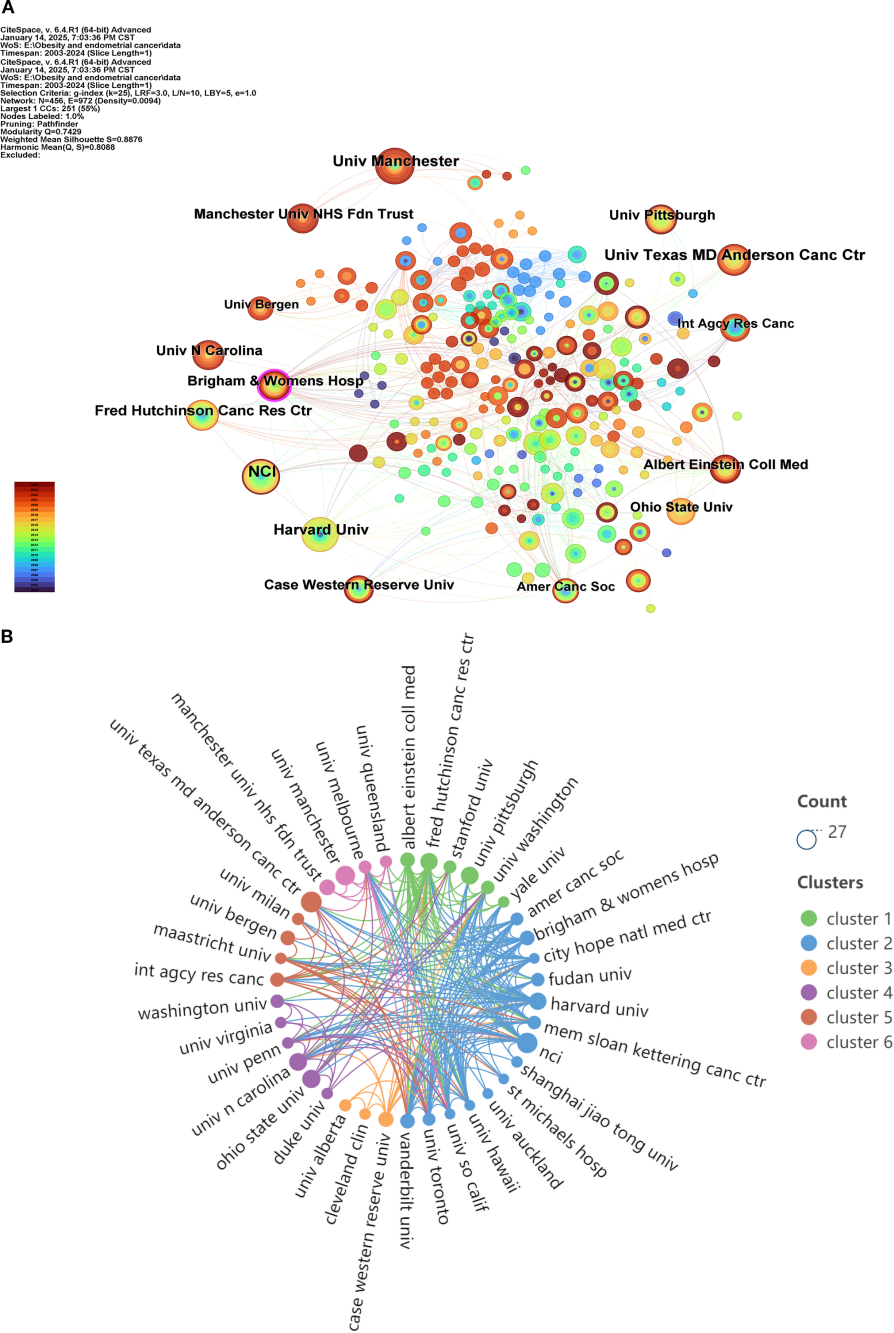
Analysis view of the issuing institution. **(A)** Co-occurrence view of the issuing institution; **(B)** View of the cooperation relationship between the issuing institution.

A total of 2,868 institutions have contributed to publications on this topic, with **Table 2** displaying the top 15 institutions ranked by publication volume. Each of these 15 institutions has published no fewer than 10 articles. The institution with the highest publication output is the NCI, which has published 23 articles, followed by the University of Texas MD Anderson Cancer Center with 21 publications. In terms of centrality, Brigham and Women’s Hospital exhibits a high centrality score of 0.21, followed by the American Cancer Society with a centrality of 0.07, indicating that the publications from these two institutions are highly authoritative and recognized by relevant stakeholders. Regarding total connection strength, NCI, Harvard University, and Fred Hutchinson Cancer Research Center rank as the top three, suggesting that these three institutions have extensive connections with others. **Figure 4** presents the co-occurrence analysis network view of these institutions, while **Figure 4B** illustrates their collaboration status, which is divided into six collaborative clusters. Within each cluster, there are strong collaborative relationships among the organizations, and there are also significant collaborative ties between the different clusters.

**Table 2:** Top 15 institutions by volume.

Rank	Count	Centrality	Institutions	Total link strength
1	23	0.05	NCI	76
2	21	0.04	Univ Texas MD Anderson Canc Ctr	23
3	19	0.01	Univ Manchester	20
4	18	0.04	Harvard Univ	73
5	16	0.01	Fred Hutchinson Canc Res Ctr	72
6	15	0.08	Albert Einstein Coll Med	52
7	15	0.21	Brigham & Womens Hosp	56
8	15	0.01	Univ Pittsburgh	11
9	14	0.03	Case Western Reserve Univ	27
10	14	0	Univ N Carolina	22
11	14	0.01	Manchester Univ NHS Fdn Trust	20
12	13	0.01	Ohio State Univ	19
13	12	0.02	Int Agcy Res Canc	45
14	11	0.07	Amer Canc Soc	39
15	10	0.04	Univ Bergen	29

### 3.4 Analysis of author publication and collaborative relationships

**Figure 5:**
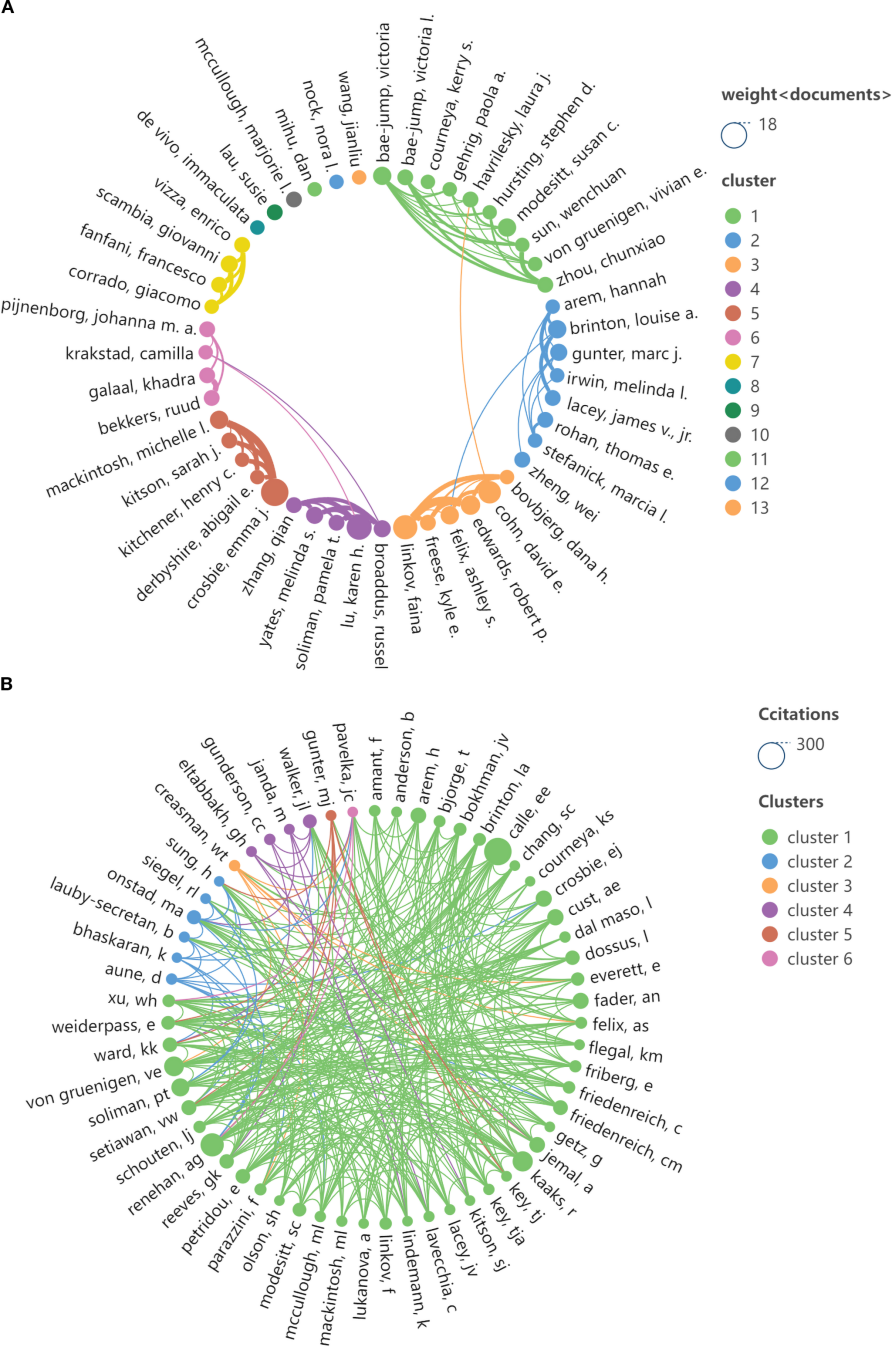
Author partnership view. **(A)** View of the cooperative relationship between authors; **(B)** The cooperative relationship view of the cited authors.

A total of 3,975 authors have contributed to publications in this field. **Table 3** presents the top 13 authors based on the volume of published literature, with each author having published no fewer than six papers. The authors with the highest number of publications are Crosbie, Emma J, and Linkov, Faina, who have published 14 and 12 works, respectively. Brinton, Louise A. has a centrality score of 0.04, ranking first among the ten scholars. **Table 4** displays the top ten authors with the highest citation counts, with each author’s articles cited at least 74 times; notably, CALLE EE’s articles have been cited the most, totaling 223 citations. Among the ten authors, SOLIMAN PT has the highest centrality score of 0.15, indicating a higher quality and authoritative nature of this scholar’s publications. **Figure 5** illustrates the collaborative network view of the authors and highly cited authors, revealing prominent clusters among these authors, indicating stable collaborative relationships. This suggests that a substantial research team has formed during the development of research on this topic.

**Table 3:** The top 10 authors by the largest number of publications.

Rank	Count	Centrality	Authors
1	14	0.02	Crosbie, Emma J
2	12	0	Linkov, Faina
3	10	0	Lu, Karen H
4	10	0.01	Gunter, Marc J
5	9	0	Cohn, David E
6	8	0.04	Brinton, Louise A
7	8	0	Edwards, Robert P
8	7	0	Bae-jump, Victoria
9	6	0	Freese, Kyle E
10	6	0	Mackintosh, Michelle L

**Table 4:** Top 10 most cited authors.

Rank	Author	Citations	Centrality
1	CALLE EE	223	0.06
2	RENEHAN AG	156	0.03
3	KAAKS R	137	0.06
4	VON GRUENIGENVE	97	0.04
5	SOLIMAN PT	91	0.15
6	CROSBIE EJ	90	0.03
7	JEMAL A	84	0.12
8	FADER AN	82	0.02
9	REEVES GK	76	0.02
10	CUST AE	74	0.12

### 3.5 Analysis of publishing journals and cited journals

**Figure 6:**
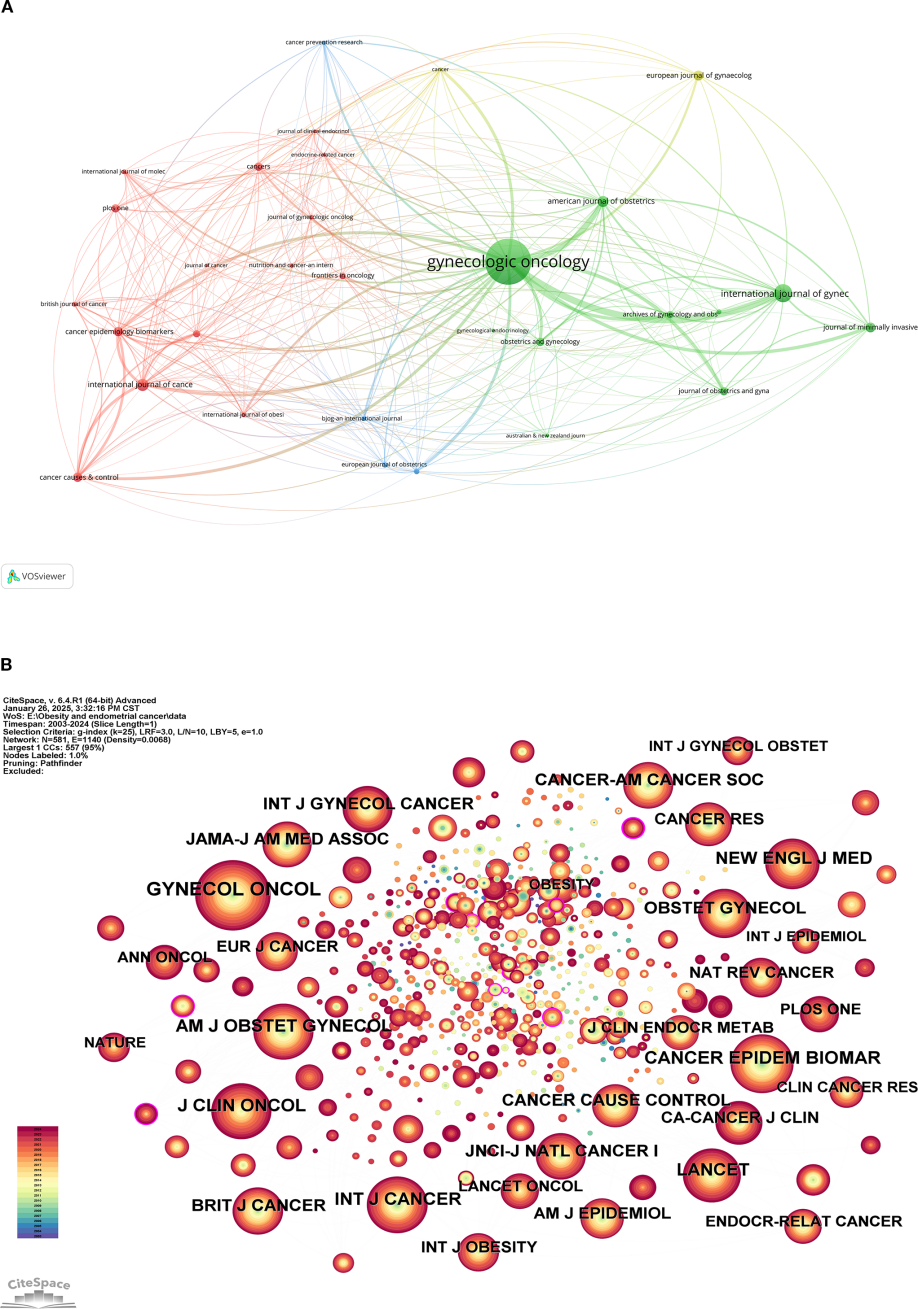
Journal network analysis view. **(A)** Network view of the published journal; **(B)** Network view of cited journals.

A total of 219 journals have published works related to this topic, with **Table 5** displaying the top 10 journals by publication volume. These 10 journals collectively published 232 articles, accounting for 34.07% of all publications. Among them, Gynecologic Oncology (Q1/4.5) had the highest number of publications (86 articles/12.63%), followed by the International Journal of Gynecological Cancer (Q1/4.5; 29 articles/4.26%) and the International Journal of Cancer (Q1/5.7; 18 articles/2.64%). **Figure 6A** presents a network view of the publishing journals, where larger node sizes indicate higher publication volumes.

**Table 5:** Top 10 journals by the number of publications.

Rank	Journal	Count	Division/IF
1	Gynecologic Oncology	86	Q1/4.5
2	International Journal of Gynecological Cancer	29	Q1/4.5
3	International Journal of Cancer	18	Q1/5.7
4	American Journal of Obstetrics and Gynecology	17	Q1/8.7
5	European Journal of Gynaecological Oncology	15	Q4/0.5
6	Journal of Minimally Invasive Gynecology	15	Q1/3.5
7	Cancer Causes & Control	14	Q3/2.2
8	Cancer Epidemiology, Biomarkers & Prevention	14	Q1/3.7
9	Cancers	12	Q1/4.5
10	Journal of Obstetrics and Gynaecology Research	12	Q2/1.6

We analyzed the cited journals, with **Table 6** showcasing the top 10 journals by citation frequency. **Figure 6B** illustrates the network analysis view of the cited journals, where larger node sizes correspond to greater publication volumes. Gynecologic Oncology (Q1/4.5; cited 535 times) is the most frequently cited journal, followed by PLOS ONE (Q1/3.7; cited 360 times) and the American Journal of Obstetrics and Gynecology (Q1/8.7; cited 348 times). In terms of centrality, the New England Journal of Medicine has the highest centrality score of 0.07, followed by Cancer Epidemiology, Biomarkers & Prevention (0.06) and the American Journal of Obstetrics and Gynecology (0.05). This indicates that the research published in these three journals has been recognized by researchers and cited multiple times.

**Table 6:** Top 10 journals with the highest number of citations.

Rank	Count	Centrality	Cited Journal	Division/IF
1	535	0	Gynecologic Oncology	Q1/4.5
2	360	0.06	Cancer Epidemiology, Biomarkers & Prevention	Q1/3.7
3	348	0.05	American Journal of Obstetrics and Gynecology	Q1/8.7
4	348	0	International Journal of Cancer	Q1/5.7
5	326	0.01	Journal of Clinical Oncology	Q1/42.1
6	321	0	Lancet	Q1/98.4
7	290	0.07	New England Journal of Medicine	Q1/96.3
8	269	0	Obstetrics & Gynecology	Q1/5.8
9	256	0	British Journal of Cancer	Q1/6.4
10	254	0.01	Cancer	Q1/6.1

**Figure 7:**
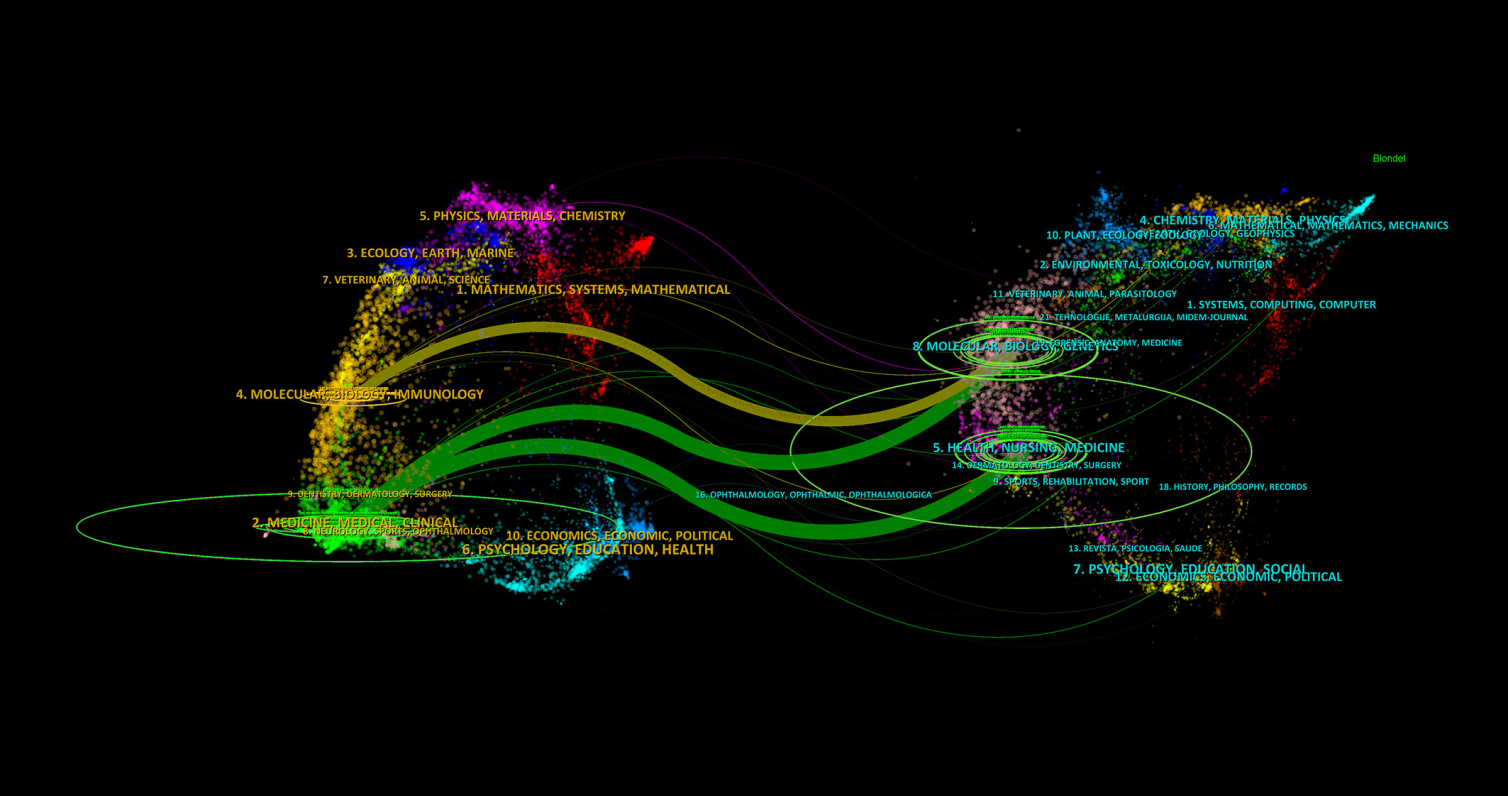
Dual mapping overlay analysis view of periodicals.

In **Figure 7**, the dual mapping overlay analysis view of the journals illustrates the distribution of topics. The labels represent the covered subject areas, while the colored paths indicate citation relationships. The analysis reveals four significant citation pathways. It is evident that articles published in journals under the “MOLECULAR, BIOLOGY, GENETICS” category are frequently cited by journals in the “MOLECULAR, BIOLOGY, IMMUNOLOGY” and “MEDICINE, MEDICAL, CLINICAL” categories; conversely, journals under the “HEALTH, NURSING, MEDICINE” category are often cited by journals in the “MEDICINE, MEDICAL, CLINICAL” category.

### 3.6 Analysis of co-cited references

**Figure 8:**
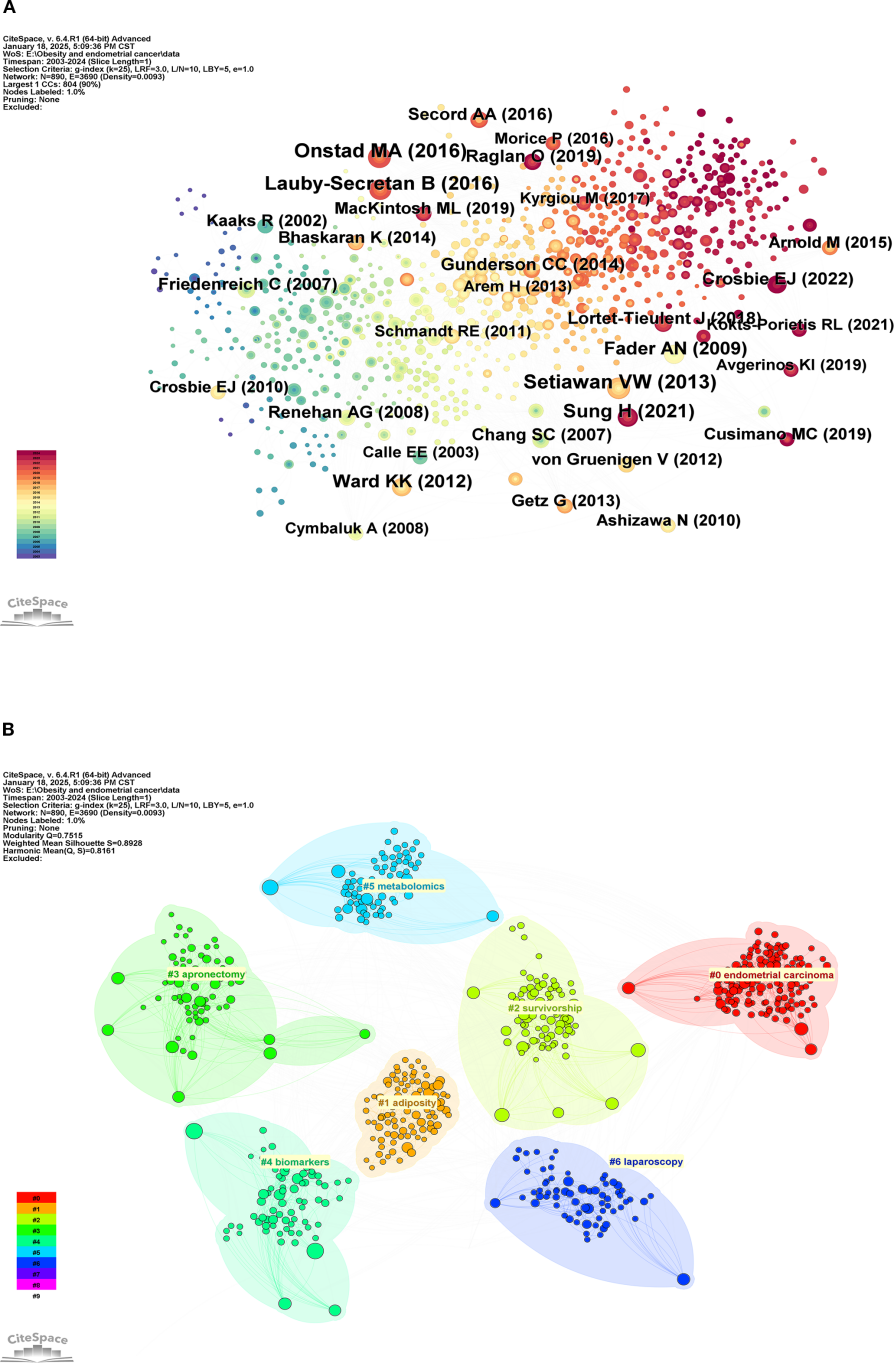
Analysis view of co-cited references. **(A)** Co-occurrence view of co-cited references; **(B)** Cluster analysis view of co-cited references.

When multiple pieces of references are cited together by different sources, a co-citation relationship is established, which is often utilized to determine the degree of association between various documents. **Table 7** presents the top ten most co-cited works (3, 8, 9, 17–23), while **Figure 8A** illustrates the co-occurrence analysis network view of these references. The most frequently cited work is “Type I and II endometrial cancers: have they different risk factors?” by Setiawan VW, published in 2013 in the Journal of Clinical Oncology. Following this is “Addressing the Role of Obesity in Endometrial Cancer Risk, Prevention, and Treatment” by Onstad MA, published in 2016 in the Journal of Clinical Oncology, and “Body Fatness and Cancer–Viewpoint of the IARC Working Group” by Lauby-Secretan B, published in 2016 in the New England Journal of Medicine. These works focus on the current research status of obesity as a risk factor for endometrial cancer. **Figure 8B** displays the network view of the clustering analysis. The results indicate that the themes of these co-cited documents primarily revolve around “endometrial carcinoma,” “adiposity,” “survivorship,” “apronectomy,” “biomarkers,” “metabolomics,” and “laparoscopy.” In the clustering analysis results, Q = 0.7515 > 0.3 and S = 0.8928 > 0.7, indicating that the clustering structure of this analysis is significant and the conclusions are compelling.

**Table 7:** The top 10 most cited references.

Rank	Count	Year	Cited Reference
1	32	2013	Setiawan VW, 2013, J CLIN ONCOL, V31, P2607, DOI 10.1200/JCO.2012.48.2596
2	32	2016	Onstad MA, 2016, J CLIN ONCOL, V34, P4225, DOI 10.1200/JCO.2016.69.4638
3	29	2016	Lauby-Secretan B, 2016, NEW ENGL J MED, V375, P794, DOI 10.1056/NEJMsr1606602
4	28	2021	Sung H, 2021, CA-CANCER J CLIN, V71, P209, DOI 10.3322/caac.21660
5	23	2009	Fader AN, 2009, GYNECOL ONCOL, V114, P121, DOI 10.1016/j.ygyno.2009.03.039
6	22	2012	Ward KK, 2012, GYNECOL ONCOL, V126, P176, DOI 10.1016/j.ygyno.2012.04.013
7	20	2022	Crosbie EJ, 2022, LANCET, V399, P1412, DOI 10.1016/S0140-6736(22)00323-3
8	20	2019	Raglan O, 2019, INT J CANCER, V145, P1719, DOI 10.1002/ijc.31961
9	19	2014	Gunderson CC, 2014, GYNECOL ONCOL, V133, P23, DOI 10.1016/j.ygyno.2014.01.041
10	18	2008	Renehan AG, 2008, LANCET, V371, P569, DOI 10.1016/S0140-6736(08)60269-X

### 3.7 Keywords analysis

#### 3.7.1 Keywords co-occurrence analysis

There are 488 keywords in 1004 publications, which reflect the focus and cohesion of research on the topic. **Table 8** shows the 15 keywords with the highest frequency, among which endometrial cancer ranks first, appearing 476 times, and body mass index ranks second, appearing 267 times. Risk, obesity, and women followed with 214, 197, and 137 appearances, respectively.

**Table 8:** The 15 keywords with the highest frequency occurrence.

Rank	Count	Centrality	Keywords
1	476	0	endometrial cancer
2	267	0.02	body mass index
3	214	0	risk
4	197	0.03	obesity
5	137	0	women
6	113	0.08	carcinoma
7	89	0.12	breast cancer
8	86	0.02	physical activity
9	81	0.05	mortality
10	70	0.05	overweight
11	67	0.06	insulin resistance
12	59	0.05	survival
13	53	0.15	expression
14	53	0.06	association
15	52	0.07	outcome

**Figure 9:**
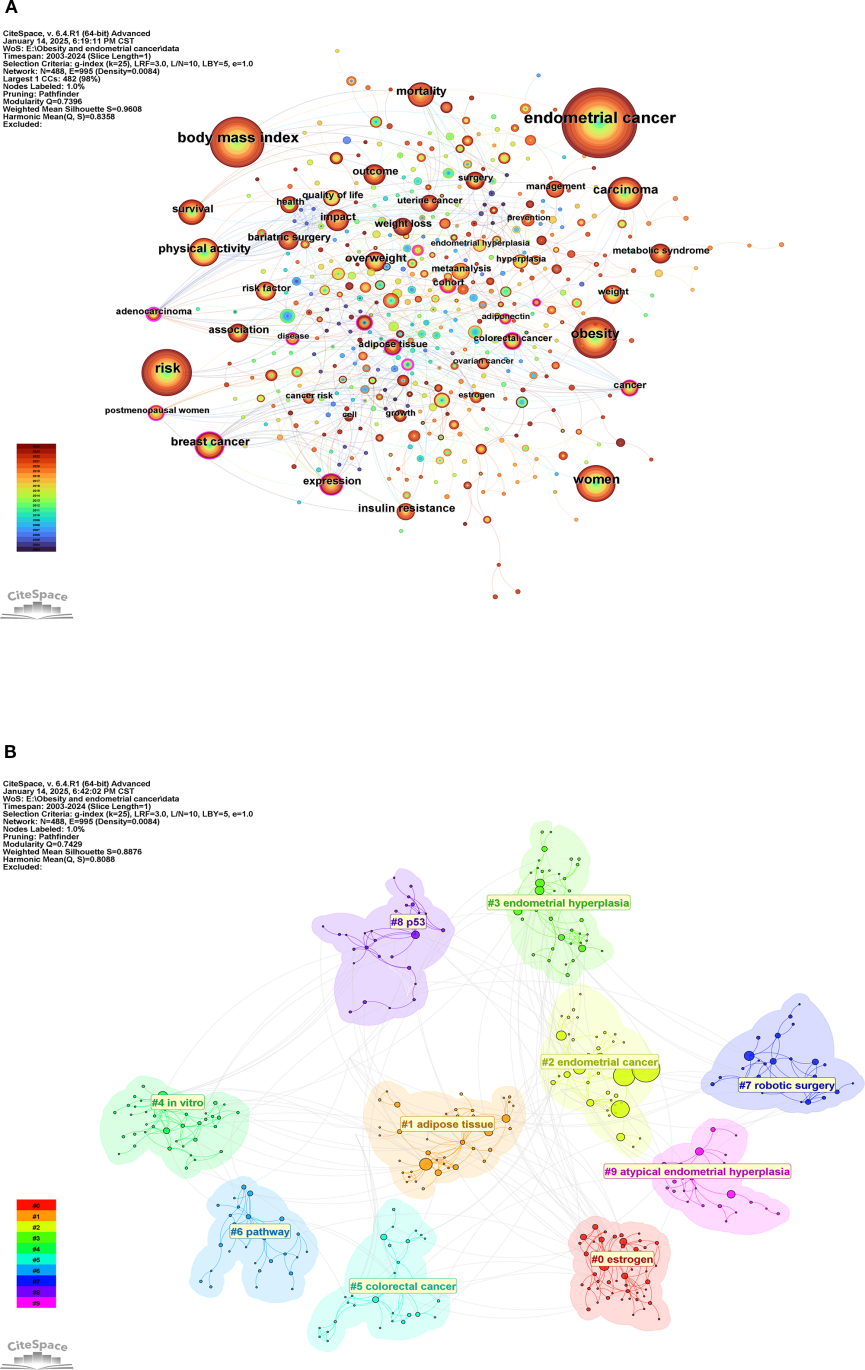
**(A)** keywords co-occurrence analysis views; **(B)** Keywords cluster analysis view.

This reflects the close association between obesity and endometrial cancer, obesity is the most important risk factor for endometrial cancer and has important reference value in the diagnosis and treatment of this disease. High-frequency keywords also include breast cancer, overweight insulin resistance, etc. These words cover diseases related to endometrial cancer and obesity-related diseases, which are the hot directions in current relevant studies. The co-occurrence network view of keywords is shown in **Figure 9A**, and the size of nodes reflects the frequency of keyword occurrence.

#### 3.7.2 Keywords clustering analysis

**Figure 9B** shows the network view of keyword clustering analysis. The analysis results show that Q = 0.7429 > 0.3, and S = 0.8878 > 0.7, indicating that the clustering structure of this analysis result is significant and the result is convincing. These keywords could be clustered into 10 clusters, among which estrogen was the most popular cluster, followed by adipose tissue, endometrial cancer, and endometrial hyperplasia. These clusters reflect the main hotspots of current research in this field, and it can be seen that scholars mainly focus on the adverse effect of obesity on endometrial cancer and endometrial-related symptoms.

#### 3.7.3 Keywords bursts analysis

**Figure 10:**
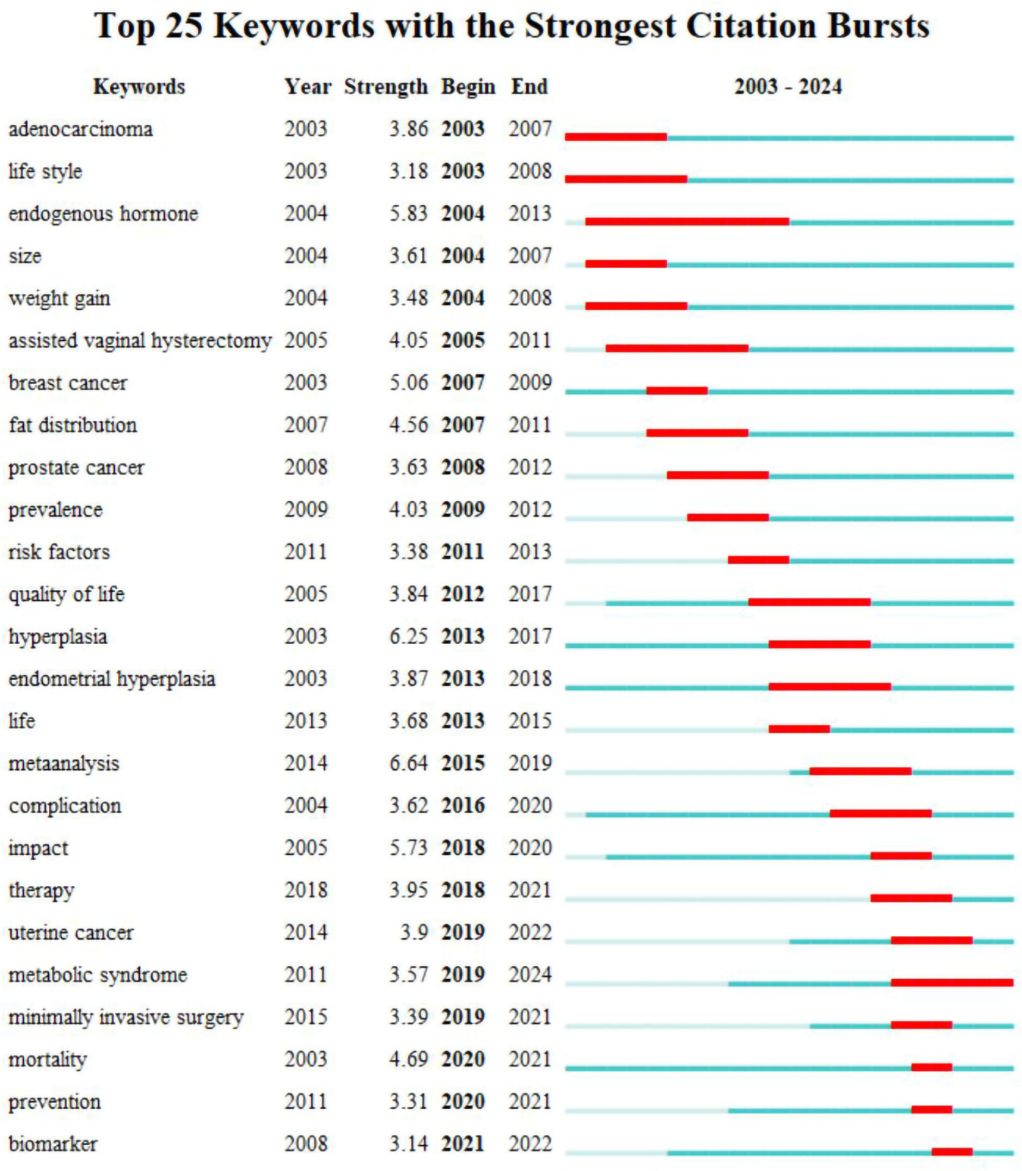
Keywords burst analysis view.

To further explore the change of research hotspots on this topic, we conducted an emergent analysis of keywords, and a total of 25 keywords were screened out and showed persistent popularity. The results are shown in **Figure 10**. adenocarcinoma and lifestyle were the first to show sustained popularity, and endogenous hormones were the longest. Metabolic syndrome has been on the rise since 2019 and continues to the present. Mortality, prevention, and biomarker are the latest three hot words, but they have not formed a long-lasting heat.

#### 3.7.4 Keywords timeline analysis

**Figure 11:**
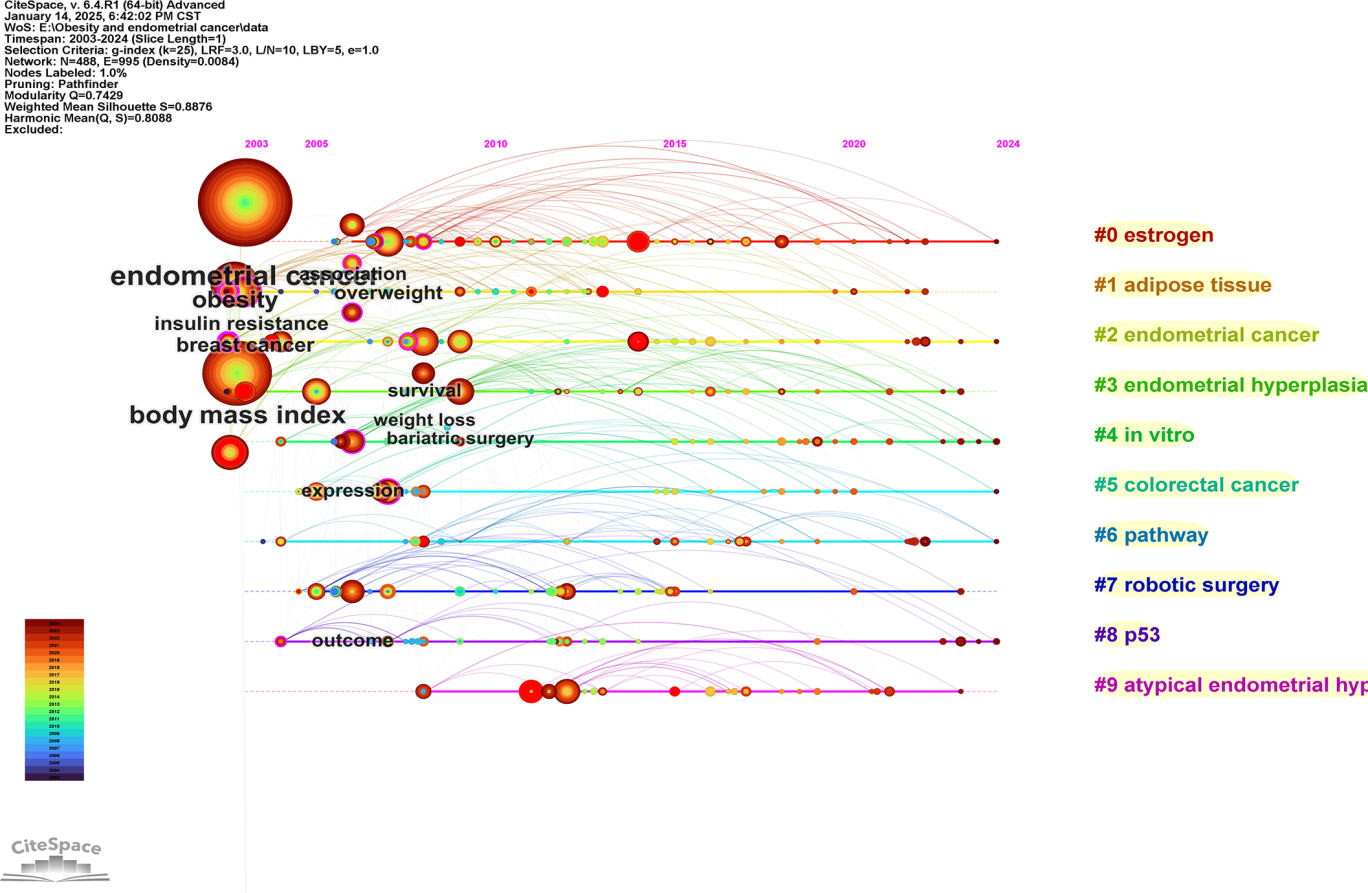
Keywords timeline analysis view.

To further analyze the heat change of keywords, we conducted a timeline analysis of keywords, as shown in **Figure 11** for details. In the timeline analysis, the keywords could be classified into 10 clusters, among which the most popular cluster “estrogen” had become a research hotspot since 2006, and endometrial was the landmark keyword in this cluster. The second most popular cluster was “adipose tissue”, which had maintained the research popularity since 2003, and obesity was the landmark keyword of this cluster. The third most popular cluster was “endometrial cancer”, and insulin resistance was the keyword of this cluster.

## 4 Discussion

### 4.1 Overview of related research on obesity and EC

In this study, we conducted bibliometric and scientometric analyses based on relevant literature concerning obesity and endocrine cancer within the WOSCC database. We systematically reviewed the current state of research in this field across multiple dimensions, including annual publication trends, country/region distribution and collaboration, institutional distribution and cooperation, author contributions and collaborative networks, journal distribution and citation patterns, co-cited literature, and keywords.

In terms of publication volume, the annual output for this theme exhibits an overall upward trajectory, peaking in 2021. Although publication numbers declined slightly between 2022 and 2024, they remained at a high level, indicating sustained academic interest in obesity-related EC research and its significant research value and developmental potential. At the national/regional level, this study encompassed 266 countries/regions across five continents, with the United States and China contributing the highest number of publications. Within international collaboration networks, particularly close cooperation was observed between the United States-China-Australia and the United Kingdom-United States-France-Netherlands axes, potentially reflecting these nations’ advantages in research resource investment and policy support.

Institutional analysis revealed that among all 2,868 research institutions, The NCIUniv Texas MD Anderson Cancer Centre led in publication output. Brigham & Women’s Hospital exhibited the highest centrality, indicating substantial influence and widespread recognition of its research. The NCI, Harvard University, and Fred Hutchinson Cancer Research Centre ranked top three in total connection strength, signifying their pivotal roles within the collaborative network and robust capacity for academic exchange. The author collaboration network encompassed 3,975 researchers, with Emma J. Crosbie and Faina Linkov publishing the most papers. Louise A. Brinton exhibited the highest centrality, while CALLE EE’s publications received the most citations. These authors formed stable and sizable collaborative groups, indicating the emergence of mature research teams within the field.

Journal analysis reveals 219 publications have featured relevant research. Gynaecologic Oncology (Q1, IF = 4.5) not only ranks highest in publication volume but also holds the most citations. The top ten journals by publication volume collectively published 232 articles, accounting for 34.07% of the total. The journal with the highest centrality among cited publications was the New England Journal of Medicine (Q1, IF = 96.3), reflecting the high authority and academic influence of its research. Cited journals predominantly clustered within the ‘MOLECULAR, BIOLOGY, GENETICS’ and ‘HEALTH, NURSING, MEDICINE’ subject domains. Analysis of the most frequently cited literature revealed that works by Setiawan VW, Onstad MA, and Lauby-Secretan B received the highest citation counts. These studies primarily explored epidemiological evidence and mechanisms linking obesity to EC risk.

For keyword analysis, “endometrial cancer” “body mass index” “risk” and “obesity” exhibited the highest frequency, reflecting current research focus on obesity as an independent risk factor for EC in screening, diagnosis and treatment. Cluster analysis identified high-frequency clusters represented by “obesity” “adipose tissue” “endometrial cancer” and “endometrial hyperplasia” indicating a concentrated trend in current research themes. Emergent term analysis indicates that ‘endogenous hormone’ maintained the longest duration of prominence, suggesting sustained interest in hormonal mechanisms within obesity-related EC. Meanwhile, “metabolic syndrome” has seen rising prominence since 2019, reflecting a recent shift in research focus towards the multifaceted interactive mechanisms between obesity-related metabolic syndrome and EC development. Although keyword analysis reveals current research hotspots and structural patterns, it also highlights a certain risk of conceptual rigidity within the field. High-frequency keywords such as “body mass index” and “obesity” continue to dominate discourse, indicating that current research predominantly centres on epidemiology and macro-level risk factors, with insufficient attention paid to underlying mechanisms, molecular pathways, or intervention strategies. The absence of emerging keywords like “immunotherapy” “microbiome” or “targeted therapy” in the clustering results suggests that future research should further expand into the molecular mechanisms of obesity-EC interactions and precision treatment domains. Moreover, while the persistent emergence of “metabolic syndrome” among prominent terms reflects interdisciplinary trends, it also calls for more research integrating metabolic factors with tumour microenvironment dynamics from multi-omics and systems biology perspectives. This approach will propel the field towards deeper insights and greater clinical translational value.

### 4.2 Research status of obesity and EC

Co-cited references usually reflects the research basis and status of a specific field (21). In this study, we explored the current status and basis of research on the relationship between obesity and EC through the most cited literature. The most cited was Setiawan VW’s “Type I and II endometrial cancers: have they different risk factors?” published in J CLIN ONCOL. “, this article pooled individual-level data from 10 cohort studies and 14 case-control studies, including 14,069 cases of endometrial cancer and 35,312 controls. We found that parity, contraceptive use, smoking, age at menarche, and diabetes were similarly associated with I and II endometrial cancers. However, BMI has a greater impact on I endometrial cancers than on II endometrial cancers (22). the second most cited was Onstad MA’s “Addressing the Role of Obesity in Endometrial Cancer Risk, Prevention,” published by J CLIN ONCOL. and Treatment “ (8). In this review, researchers identify the molecular mechanisms by which obesity and adipose tissue contribute to the development of endometrial cancer, further discuss the impact of obesity on the clinical management of the disease, and examine advances in rational behavioral and pharmacologic interventions to reduce the risk of endometrial cancer and improve cancer outcomes. And to preserve fertility in increasingly younger women with endometrial cancer. In third place was Lauby-Secretan B’s “Body Fatness and Cancer–Viewpoint of the IARC Working Group” published in NEW ENGL J MED, in which, Researchers identified the cellular and molecular mechanisms associated with obesity during carcinogenesis and assessed the relevance of each mechanism to cancer as well as to specific organ sites. It has been proposed that weight loss has a positive effect on these mechanisms (23). Fader AN’s 2009 study reviewed epidemiological trends in obesity and endometrial cancer, discussed the promising role of screening biomarker studies, reviewed prevention efforts and modifiable risk factors, and approaches to optimize health outcomes and quality of life in endometrial cancer survivors (24). Crosbie EJ’s study published in LANCET proposes that the rising prevalence of obesity is the main underlying cause of EC. Obesity poses a challenge to the diagnosis and treatment of EC, and more research is needed to provide primary prevention and optimize endometrial cancer survival in high-risk women (25). Raglan O’s study summarized risk factors for endometrial cancer and proposed that body mass index and waist-to-hip ratio were associated with an increased risk of cancer and total endometrial cancer in premenopausal women (26). The study by Gunderson CC, which analyzed the effect of obesity on surgical staging, complications, and survival of endometrial cancer, suggested that obese women have a greater risk of surgery and a lower risk of metastatic disease. BMI was associated with all-cause mortality but not disease-specific mortality, underscoring the deleterious effects of obesity (independent of EC) that deserve special attention (27).

The research on the mechanism between obesity and EC has made positive progress, and visceral fat plays a key role in this process. As a complex endocrine organ, visceral fat is composed of adipocytes, preadipocytes, infiltrating macrophages, stroma, nerves, and stem cells that collectively secrete adipokines that increase endometrial proliferation and promote tumor gene expression (28–30). In premenopausal women, cyclic expression of estrogen drives endometrial proliferation (31), and after menopause, adipose tissue becomes the primary site of estrogen synthesis (32). The main sources of aromatase, the enzyme responsible for the conversion of androgens to estrogens, are adipocytes, preadipocytes, and mesenchymal stem cells within adipose tissue (33, 34). Aromatase expression levels and activity increase with age and obesity status (34, 35). Thus, obesity promotes estrogen-induced endometrial proliferation in postmenopausal women (36, 37). Based on the impact of obesity on EC, reducing the overall prevalence of obesity may have a positive effect on reducing EC incidence, but corresponding data to support this are still lacking. Bariatric surgery can result in significant weight loss, and the results of a meta-analysis showed a 60% reduction in the probability of EC in patients who underwent bariatric surgery compared with obese controls (38). In clinical treatment, obesity can complicate the clinical management strategy of EC. The main treatment for patients with early-stage EC includes hysterectomy and removal of bilateral fallopian tubes and ovaries, which is difficult in obese patients because of their body size. In addition, complications of obesity can also put EC patients at higher surgical risk (39). Thus, robotic surgery may offer advantages over traditional surgical approaches (40). For premenopausal young EC patients, ovarian preservation and fertility should be considered as much as possible. Preoperative MRI imaging is important to rule out myometrial invasion and synchronous ovarian tumors. Strategies for clinical management should be explored to better define the role of weight loss, diet, and exercise in improving the disease-specific and overall survival rates of EC survivors. Furthermore, individualized treatment plans should be developed according to the specific conditions of patients to improve the clinical outcomes of EC patients.

Combined with this high-impact literature, it is not difficult to find that scholars have found a potentially close relationship between obesity and EC. Their research focuses on exploring the mechanism of association between obesity and EC, analyzing the impact of obesity on clinical treatment strategies of EC, and aiming to improve disease prevention and treatment strategies.

### 4.3 Future trends in obesity and EC research

Keywords can reflect the overall theme of research in the field, and the analysis of keywords helps to identify important themes and future research trends in the field (41). Co-occurrence analysis of keywords found that endometrial cancer was the most frequently used keyword, followed by body mass index, risk, obesity, and women. High-frequency keywords also included EC-related diseases or symptoms such as carcinoma, breast cancer, cancer, and insulin resistance. Keywords: overweight, weight loss, weight, and adipose tissue; Other high-frequency keywords included bariatric surgery, quality of life, and risk factor. It can be seen that the important topics of current research focus on EC and related diseases, as well as related research on weight as a risk factor for EC. Obesity surgery and the quality of life of patients have also attracted the attention of scholars. Keyword cluster analysis showed that the hot clusters were concentrated in the following aspects: ① related diseases: endometrial cancer, colorectal cancer, and endometrial hyperplasia; ② Detection techniques and indicators: estrogen, p53; ③ *in vitro*, robotic surgery; ④ Pathological factors and mechanisms: adipose tissue, pathway. This reflects future research trends, colorectal cancer and endometrial hyperplasia are EC-related diseases that scholars pay more attention to; estrogen and adipose tissue can be seen that scholars have explored the correlation mechanism between hormones and EC; *in vitro* and robotic surgery responds to the interventions to deal with obesity. The emergence analysis of keywords helped find the changes in research topics on this topic. Mortality, prevention, and biomarker were the three latest hotspots. Metabolic syndrome showed a hot trend from 2019 and continues to the present. According to the timeline analysis, endometrial cancer, obesity, and body mass index were the most significant keywords in these hot spot clusters. Compared to other studies of the same type, this study has thoroughly analyzed the data from authoritative databases through several software to draw reliable conclusions.

In recent years, multidisciplinary convergence—particularly the synergy between molecular biology, imaging science and epidemiology—has substantially advanced research into EC and its associated conditions. Regarding diagnosis, while conventional tumour marker testing, cytology and pathological biopsy remain the cornerstones of EC diagnosis, emerging technologies are rapidly expanding the frontiers of early diagnosis and non-invasive monitoring. For instance, liquid biopsy offers minimally invasive approaches for early EC detection and recurrence monitoring by detecting circulating tumour DNA, RNA, or exosomes as biomarkers. Particularly noteworthy is the introduction of artificial intelligence-assisted diagnostic technologies, which significantly enhance the accuracy and efficiency of imaging and pathological data processing, laying the groundwork for personalised prediction and precision diagnosis. On the therapeutic front, targeted drugs and immunotherapy have brought renewed hope to EC patients. Concurrently, advances in novel drug delivery systems and minimally invasive surgical techniques have further enhanced treatment efficacy while reducing patient trauma. These clinical advancements rely heavily on the support of preclinical models (such as organoids and transgenic animal models) and standardised clinical trials, collectively forming a crucial pathway for translating EC treatments from basic research to clinical application.

Looking ahead, given the strong epidemiological and biological link between obesity and EC, several cutting-edge technologies are poised to play pivotal roles. Single-cell omics and multi-omics integration analyses (proteomics, metabolomics, etc.) can delve into the mechanisms underpinning tumour microenvironment heterogeneity and obesity-related metabolic dysregulation in EC pathogenesis. Furthermore, gene editing technologies offer powerful tools for functional validation, potentially identifying novel therapeutic targets. In summary, artificial intelligence, multi-omics technologies, and interdisciplinary collaboration will collectively shape new paradigms in EC research—particularly regarding obesity-associated EC—driving the field towards more precise, dynamic, and personalised approaches.

## 5 Conclusion

Our study found that scholars have found a potentially close relationship between obesity and EC. Current research focuses on exploring the mechanism of association between obesity and EC, analyzing the impact of obesity on clinical treatment strategies of EC, and aiming to improve disease prevention and treatment strategies. There are several directions for future research: (1) the scope of research will be expanded to EC-related diseases; (2) Typical indicators and diagnostic techniques of EC will be paid more attention; (3) Clinical workers should focus on the development of new treatment methods and techniques to improve clinical efficacy; (4) Researchers should further strengthen the exploration of the pathological mechanism of obesity and EC, and clarify the specific pathways of obesity affecting EC.The formation of multidisciplinary teams, the rational application of diagnostic and therapeutic techniques, the further strengthening of the exploration of pathological mechanisms related to obesity and cardiovascular disease, and the improvement of clinical diagnostic and therapeutic strategies are powerful measures to promote the development of this field.

## 6 Strengths and limitations of this study

This study employed bibliometric methods to systematically review the overall research landscape concerning obesity and EC-related fields, summarising current research hotspots and projecting future trends to provide objective analysis and conclusions for this domain. Nevertheless, certain limitations remain. Firstly, data was sourced exclusively from the Web of Science Citation Citation (WOSCC) database, potentially resulting in the exclusion of relevant literature and affecting the comprehensiveness of analytical outcomes. Secondly, the exclusion of non-English publications introduces potential language bias, potentially underrepresenting research outcomes from specific regions or with cultural specificity. This limits the global representativeness and universal applicability of the conclusions. Despite these data source constraints, this study offers valuable insights based on existing publicly available data and lays the groundwork for future, broader analyses incorporating data from multiple languages.

## Data Availability

The original contributions presented in the study are included in the article/supplementary material, further inquiries can be directed to the corresponding author/s.

## References

[1] Clarke MA Devesa SS Hammer A Wentzensen N. Racial and ethnic differences in hysterectomy-corrected uterine corpus cancer mortality by stage and histologic subtype. JAMA Oncol. (2022) 8:895–903. doi: 10.1001/jamaoncol.2022.0009, PMID: 35511145 PMC9073658

[2] Zheng W Lin X Chen H Yang Z Zhao H Li S. Gut microbiota and endometrial cancer: research progress on the pathogenesis and application. Ann Med. (2025) 57:2451766. doi: 10.1080/07853890.2025.2451766, PMID: 39810645 PMC11737052

[3] Sung H Ferlay J Siegel RL Laversanne M Soerjomataram I Jemal A. Global cancer statistics 2020: GLOBOCAN estimates of incidence and mortality worldwide for 36 cancers in 185 countries. CA: Cancer J Clin. (2021) 71:209–49. doi: 10.3322/caac.21660, PMID: 33538338

[4] Siegel RL Miller KD Wagle NS Jemal A. Cancer statistics, 2023. CA: Cancer J Clin. (2023) 73:17–48. doi: 10.3322/caac.21763, PMID: 36633525

[5] Torre LA Islami F Siegel RL Ward EM Jemal A. Global cancer in women: burden and trends. Cancer Epidemiol Biomarkers Prev. (2017) 26:444–57. doi: 10.1158/1055-9965.EPI-16-0858, PMID: 28223433

[6] Centini G Colombi I Ianes I Perelli F Ginetti A Cannoni A. Fertility sparing in endometrial cancer: where are we now? Cancers. (2025) 17:112. doi: 10.3390/cancers17010112, PMID: 39796739 PMC11720406

[7] Sakaue T Dorayappan KDP Zingarelli R Khadraoui W Anbalagan M Wallbillich J. Obesity-induced extracellular vesicles proteins drive the endometrial cancer pathogenesis: therapeutic potential of HO-3867 and Metformin. Oncogene. (2024) 43:3586–97. doi: 10.1038/s41388-024-03182-2, PMID: 39414985 PMC11602708

[8] Onstad MA Schmandt RE Lu KH. Addressing the role of obesity in endometrial cancer risk, prevention, and treatment. J Clin Oncol. (2016) 34:4225–30. doi: 10.1200/JCO.2016.69.4638, PMID: 27903150 PMC5455320

[9] Renehan AG Tyson M Egger M Heller RF Zwahlen M. Body-mass index and incidence of cancer: a systematic review and meta-analysis of prospective observational studies. Lancet (London England). (2008) 371:569–78. doi: 10.1016/S0140-6736(08)60269-X, PMID: 18280327

[10] Aune D Navarro Rosenblatt DA Chan DS Vingeliene S Abar L Vieira AR. Anthropometric factors and endometrial cancer risk: a systematic review and dose-response meta-analysis of prospective studies. Ann Oncol. (2015) 26:1635–48. doi: 10.1093/annonc/mdv142, PMID: 25791635

[11] Crosbie EJ Zwahlen M Kitchener HC Egger M Renehan AG. Body mass index, hormone replacement therapy, and endometrial cancer risk: a meta-analysis. Cancer Epidemiol Biomarkers Prev. (2010) 19:3119–30. doi: 10.1158/1055-9965.EPI-10-0832, PMID: 21030602

[12] von Gruenigen VE Tian C Frasure H Waggoner S Keys H Barakat RR. Treatment effects, disease recurrence, and survival in obese women with early endometrial carcinoma: a Gynecologic Oncology Group study. Cancer. (2006) 107:2786–91. doi: 10.1002/cncr.22351, PMID: 17096437

[13] Poston L Caleyachetty R Cnattingius S Corvalán C Uauy R Herring S. Preconceptional and maternal obesity: epidemiology and health consequences. Lancet Diabetes Endocrinol. (2016) 4:1025–36. doi: 10.1016/S2213-8587(16)30217-0, PMID: 27743975

[14] Naqvi A MacKintosh ML Derbyshire AE Tsakiroglou AM Walker TDJ McVey RJ. The impact of obesity and bariatric surgery on the immune microenvironment of the endometrium. Int J Obes (2005). (2022) 46:605–12. doi: 10.1038/s41366-021-01027-6, PMID: 34857870 PMC8872994

[15] Hussain SP Hofseth LJ Harris CC. Radical causes of cancer. Nat Rev Cancer. (2003) 3:276–85. doi: 10.1038/nrc1046, PMID: 12671666

[16] Ames BN Gold LS Willett WC. The causes and prevention of cancer. Proc Natl Acad Sci U States A. (1995) 92:5258–65. doi: 10.1073/pnas.92.12.5258, PMID: 7777494 PMC41674

[17] Balkwill F Coussens LM. Cancer: an inflammatory link. Nature. (2004) 431:405–6. doi: 10.1038/431405a, PMID: 15385993

[18] Ninkov A Frank JR Maggio LA. Bibliometrics: Methods for studying academic publishing. Perspect Med Educ. (2022) 11:173–6. doi: 10.1007/S40037-021-00695-4, PMID: 34914027 PMC9240160

[19] Cooper ID. Bibliometrics basics. J Med Library Assoc: JMLA. (2015) 103:217–8. doi: 10.3163/1536-5050.103.4.013, PMID: 26512226 PMC4613387

[20] Chen C Song M. Visualizing a field of research: A methodology of systematic scientometric reviews. PloS One. (2019) 14:e0223994. doi: 10.1371/journal.pone.0223994, PMID: 31671124 PMC6822756

[21] Liu Y Sun D Huang Y Shen Y Chen T Chen W. Bibliometric analysis of research on retinoic acid in the field of kidney disorders. Front Pharmacol. (2024) 15:1435889. doi: 10.3389/fphar.2024.1435889, PMID: 39211779 PMC11357955

[22] Setiawan VW Yang HP Pike MC McCann SE Yu H Xiang YB. Type I and II endometrial cancers: have they different risk factors? J Clin Oncol. (2013) 31:2607–18. doi: 10.1200/JCO.2012.48.2596, PMID: 23733771 PMC3699726

[23] Lauby-Secretan B Scoccianti C Loomis D Grosse Y Bianchini F Straif K. Body fatness and cancer–viewpoint of the IARC working group. New Engl J Med. (2016) 375:794–8. doi: 10.1056/NEJMsr1606602, PMID: 27557308 PMC6754861

[24] Fader AN Arriba LN Frasure HE von Gruenigen VE. Endometrial cancer and obesity: epidemiology, biomarkers, prevention and survivorship. Gynecol Oncol. (2009) 114:121–7. doi: 10.1016/j.ygyno.2009.03.039, PMID: 19406460

[25] Crosbie EJ Kitson SJ McAlpine JN Mukhopadhyay A Powell ME Singh N. Endometrial cancer. Lancet (London England). (2022) 399:1412–28. doi: 10.1016/S0140-6736(22)00323-3, PMID: 35397864

[26] Raglan O Kalliala I Markozannes G Cividini S Gunter MJ Nautiyal J. Risk factors for endometrial cancer: An umbrella review of the literature. Int J Cancer. (2019) 145:1719–30. doi: 10.1002/ijc.31961, PMID: 30387875

[27] Gunderson CC Java J Moore KN Walker JL. The impact of obesity on surgical staging, complications, and survival with uterine cancer: a Gynecologic Oncology Group LAP2 ancillary data study. Gynecol Oncol. (2014) 133:23–7. doi: 10.1016/j.ygyno.2014.01.041, PMID: 24680587

[28] Renehan AG Zwahlen M Egger M. Adiposity and cancer risk: new mechanistic insights from epidemiology. Nat Rev Cancer. (2015) 15:484–98. doi: 10.1038/nrc3967, PMID: 26205341

[29] Allott EH Hursting SD. Obesity and cancer: mechanistic insights from transdisciplinary studies. Endocr Relat Cancer. (2015) 22:R365–86. doi: 10.1530/ERC-15-0400, PMID: 26373570 PMC4631382

[30] Park J Morley TS Kim M Clegg DJ Scherer PE. Obesity and cancer–mechanisms underlying tumour progression and recurrence. Nat Rev Endocrinol. (2014) 10:455–65. doi: 10.1038/nrendo.2014.94, PMID: 24935119 PMC4374431

[31] Mihm M Gangooly S Muttukrishna S. The normal menstrual cycle in women. Anim Reprod Sci. (2011) 124:229–36. doi: 10.1016/j.anireprosci.2010.08.030, PMID: 20869180

[32] Davis SR Lambrinoudaki I Lumsden M Mishra GD Pal L Rees M. Menopause. Nat Rev Dis Primers. (2015) 1:15004. doi: 10.1038/nrdp.2015.4, PMID: 27188659

[33] Blakemore J Naftolin F. Aromatase: contributions to physiology and disease in women and men. Physiol (Bethesda Md). (2016) 31:258–69. doi: 10.1152/physiol.00054.2015, PMID: 27252161

[34] O’Connor KA Ferrell RJ Brindle E Shofer J Holman DJ Miller RC. Total and unopposed estrogen exposure across stages of the transition to menopause. Cancer Epidemiol Biomarkers Prev. (2009) 18:828–36. doi: 10.1158/1055-9965.EPI-08-0996, PMID: 19240232 PMC2675575

[35] Bulun SE Simpson ER. Regulation of aromatase expression in human tissues. Breast Cancer Res Treat. (1994) 30:19–29. doi: 10.1007/BF00682738, PMID: 7949202

[36] Vicennati V Garelli S Rinaldi E Rosetti S Zavatta G Pagotto U. Obesity-related proliferative diseases: the interaction between adipose tissue and estrogens in post-menopausal women. Hormone Mol Biol Clin Invest. (2015) 21:75–87. doi: 10.1515/hmbci-2015-0002, PMID: 25781553

[37] Zhao H Zhou L Shangguan AJ Bulun SE. Aromatase expression and regulation in breast and endometrial cancer. J Mol Endocrinol. (2016) 57:R19–33. doi: 10.1530/JME-15-0310, PMID: 27067638 PMC5519084

[38] Upala S Sanguankeo A. Bariatric surgery reduces risk of endometrial cancer. Surg Obes Relat Dis. (2015) 11:1410. doi: 10.1016/j.soard.2015.08.501, PMID: 26507941

[39] Bouwman F Smits A Lopes A Das N Pollard A Massuger L. The impact of BMI on surgical complications and outcomes in endometrial cancer surgery–an institutional study and systematic review of the literature. Gynecol Oncol. (2015) 139:369–76. doi: 10.1016/j.ygyno.2015.09.020, PMID: 26407479

[40] Gehrig PA Cantrell LA Shafer A Abaid LN Mendivil A Boggess JF. What is the optimal minimally invasive surgical procedure for endometrial cancer staging in the obese and morbidly obese woman? Gynecol Oncol. (2008) 111:41–5. doi: 10.1016/j.ygyno.2008.06.030, PMID: 18694588

[41] Dos Santos JRN Alves ICB Marques ALB Marques EP. Bibliometric analysis of global research progress on electrochemical degradation of organic pollutants. Environ Sci pollut Res Int. (2022) 29:54769–81. doi: 10.1007/s11356-022-19534-y, PMID: 35305220 PMC8934053

